# The FBI1/Akirin2 Target Gene, BCAM, Acts as a Suppressive Oncogene

**DOI:** 10.1371/journal.pone.0078716

**Published:** 2013-11-06

**Authors:** Hirotada Akiyama, Yoshimasa Iwahana, Mikiya Suda, Atsunori Yoshimura, Hiroyuki Kogai, Ai Nagashima, Hiroko Ohtsuka, Yuko Komiya, Fumio Tashiro

**Affiliations:** Department of Biological Science and Technology, Faculty of Industrial Science and Technology, Tokyo University of Science, Katsushika-ku, Tokyo, Japan; The University of Hong Kong, China

## Abstract

Basal cell adhesion molecule (BCAM), known to be a splicing variant of Lutheran glycoprotein (LU), is an immunoglobulin superfamily membrane protein that acts as a laminin α5 receptor. The high affinity of BCAM/LU for laminin α5 is thought to contribute to the pathogenesis of sickle red blood cells and to various developmental processes. However, the function of BCAM in carcinogenesis is poorly understood. Based on microarray expression analysis, we found that BCAM was one of the target genes of the oncogenic 14-3-3β-FBI1/Akirin2 complex, which acts as a transcriptional repressor and suppresses MAPK phosphatase-1 gene expression. To elucidate the detailed function of BCAM in malignant tumors, we established BCAM-expressing hepatoma K2 cells. These cells lost the malignant characteristics of parental cells, such as anchorage-independent growth, migration, invasion, and tumorigenicity. Moreover, luciferase reporter assays and chromatin immunoprecipitation analysis revealed that the 14-3-3β-FBI1/Akirin2 complex bound to the *BCAM* promoter and repressed transcription. Thus, these data indicate that BCAM is a suppressive oncoprotein, and that FBI1/Akirin2 is involved in tumorigenicity and metastasis of hepatoma through the downregulation of suppressive oncogenes.

## Introduction

14-3-3 proteins regulate many cellular processes, including the cell cycle, metabolism, signal transduction, malignant transformation, and apoptosis. We previously reported that 14-3-3β is implicated in the positive regulation of cell cycle progression and tumorigenesis [1]. 14-3-3β is over-expressed in various cancer cell lines, including aflatoxin B1 (AFB1)-induced rat hepatocellular carcinoma K1 and K2 cells [2], [3]. Enforced expression of antisense *14-3-3*β inhibits cell proliferation, colony formation in soft agar, tumorigenicity, and angiogenesis of rat hepatoma K2 cells. Furthermore, overexpression of 14-3-3β in NIH3T3 cells confers tumorigenicity in nude mice via activation of the mitogen-activated protein kinase (MAPK) cascade. Mutations in *ras* family oncogenes and suppressive oncogenes such as *p53* and *Rb* are not detected in K2 cells [4], [5]. Therefore, it is most likely that 14-3-3β plays an important role in the malignancy of K2 cells.

To further analyze the oncogenic function of 14-3-3β, we screened for 14-3-3β binding partners by the yeast two-hybrid system using 14-3-3β as a bait [6], [7]. The novel 14-3-3β binding factor, fourteen-three-three beta interactant 1 (FBI1), also known as Akirin2, plays a crucial role in tumorigenicity and lung metastasis in K2 cells. FBI1/Akirin2 promotes sustained ERK1/2 activation through repression of *mitogen-activated protein kinase phosphatase 1 (MKP-1)* transcription, resulting in the promotion of tumorigenicity and metastasis [6], [7]. Furthermore, to examine the function of FBI1/Akirin2 as a transcriptional repressor and to identify its target genes, a microarray experiment compared parental K2 cells with stable knockdown K2 cells of FBI1/Akirin2 [8]. We identified the *basal cell adhesion molecule* (*BCAM*) gene as one of the genes downregulated by FBI1/Akirin2.

BCAM, known to be a splicing variant of Lutheran glycoprotein (LU), is an immunoglobin superfamily membrane protein and acts as a laminin α5 receptor [9]. BCAM's role as a laminin receptor is presumed to play a potential role in malignant and metastatic tumors because of evidence suggesting that laminin interacts with tumor cells to promote malignant and metastatic phenotypes [10]–[12]. In fact, there are reports that BCAM affects tumor development. BCAM is induced in epithelial skin tumors [10], and haptotactic migration of BCAM overexpressing NIH3T3 cells is significantly increased on the laminin matrix [11]. Moreover, laminin α5 and BCAM are broadly expressed in HCC cells, and HCC cells attach to laminin containing the α5 chain more so than primary hepatocytes [12]. Thus, adhesive interaction of BCAM and laminin contributes to the progression of some tumors. However, there is no direct evidence that it affects tumorigenicity, and the issue still remains obscure. In addition, the expression level of BCAM mRNA in FBI1 knockdown cells is estimated to be 6-fold higher than in control cells, and this increase was the highest among identified genes by microarray analysis [8]. Therefore, we analyzed the tumorigenic role of BCAM, which is an FBI1/Akirin2 target gene. Our results suggest that BCAM functions as a tumor suppressor in rat hepatocellular carcinoma K2 cells.

## Materials and Methods

### Cell culture

K2 cells established from AFB_1_-induced rat hepatoma were cultivated with Dulbecco's modified Eagle's medium (DMEM) supplemented with 5% fetal calf serum (FCS). Cells were maintained at 37°C in a humidified atmosphere of 5% CO_2_ in air (2).

### RNA isolation and northern blot analysis

Total RNAs were isolated from various cell lines by the acidic guanidine thiocyanate/phenol/chloroform method [13]. Aliquots (20 µg) of total RNAs were electrophoresed in a 1% agarose-formaldehyde gel, transferred to a Hybond-N+ membrane (GE Healthcare), and the filter was hybridized with ^32^P-labelled probes as previously described [14]. Probes consisted of the 1.1-kb *Not*I-*Bam*HI fragment of full-length *BCAM*, and the 0.7-kb *Not*I-*Xho*I fragment of FBI1/Akirin2.

### RT-PCR

Total RNAs were prepared from various cell lines as described above. Reverse transcription (RT) reactions were performed using total RNAs and Moloney murine leukemia virus reverse transcriptase, according to the manufacturer's instructions (Invitrogen). The RT reaction mixtures were subjected to PCR amplification using specific primers as follows: mmp9-forward, 5′-TGG CTC TAG GCT ACA GCT TTG CTG C-3′, mmp9-reverse, 5′-CGA AGG AGT CAT CGA TCA CGT GTC G-3′; cyclinD1-forward, 5′-CTC CAT GTT CCA AAA CCA TTC C-3′, cyclinD1-reverse, 5′-GGG CAA CCT TCC CAA TAA ATA C-3′; FBI1-forward, 5′-TGG ATT TCG ACC CAC TGC TTA GC-3′, FBI1-reverse, 5′-GAT CAT CCC AAC CTG CCT TAG AG-3′; mkp1-forward, 5′-CCA TGG TGA TGG AGG TGG GCA TCC T-3′, mkp1-reverse, 5′-CCT TCA GCA GCT CGG AGA GGT TGT G-3′.

### Stable transfectants

BCAM expression vector was constructed by the insertion of a BCAM open reading fragment into the pcDNA3 expression vector. K2 cells were transfected with BCAM expression vector or empty vector using Lipofectamine regent (Invitrogen) according to the manufacturer's instructions. After 2 weeks of selection with 1 mg/ml G418, resistant clones were expanded and analyzed for the expression level of BCAM by western blotting.

### Western blot analysis

Western blotting was performed as described previously [15]. Anti-14-3-3β and anti-BCAM antibodies (1∶1000; Santa Cruz Biotechnology, Inc.), anti-FBI1/Akirin2 antibody (1∶1000; ProSci Inc.), and anti-Actin (1∶2000; Sigma) were used.

### Growth and colonization *in vitro*

Cells (2×10^4^) were plated in 24-well plates containing DMEM supplemented with 5% FCS and cultivated for various times. The cell number was counted using a hemocytometer. For soft agar assays, cells (1×10^3^) were suspended in 0.3% agar medium containing 5% FCS and layered on a 0.5% agar-coated 35-mm dish and cultivated for 2 weeks. The colonies formed were stained with 0.25% 1-p-iodophenyl-p-nitrophenyl-5-phenyltetrazolium chloride (INT, Sigma) for 12 h, and the number of colonies (>0.2 mm in diameter) was counted [16].

### Wound healing assay

A “wound” was introduced into a confluent monolayer of K2 cells with a sterile tip of a micropipette, and the culture medium was replaced with fresh medium. The wound area was photographed with phase contrast at marked positions (three different fields per well in triplicate). Cells were allowed to migrate for 12 and 24 h at 37°C, and the same fields were photographed again. Scratched areas were measured with ImageJ software (NIH, Bethesda, USA), and recovered surface areas over 6, 12, and 24 h were calculated compared to control cells. Photographs of the wounded areas were taken at different times after wounding the monolayer.

### Matrigel invasion assay

For the invasion assay, 6×10^4^ cells were plated on the top chamber with a Matrigel-coated membrane (24-well insert; pore size, 8 µm) (BD Biosciences, Bedford, MA) in medium with 0.1% FCS, and medium supplemented with 10% FCS was used as a chemoattractant in the lower chamber. After 48 h, cells on the lower surface of the membrane were stained with crystal violet and counted.

### Tumorigenicity in SCID mice

Cells (5×10^4^/200 µl of phosphate-buffered saline/flank) were inoculated subcutaneously into 6-week-old SCID mice (NOD.CB17-Prkdc^scid^/J; Jackson Laboratory). Tumor volume was calculated according to the formula *V* = *a*×*b* ^2^×0.52, where *a* is the largest diameter and *b* the smallest diameter of the tumor. The average volumes of the tumors were represented by the mean tumor value ± SE (*n* = 5).

### Ethics statement

Mouse care and handling conformed to the National Institutes of Health guidelines for animal research. The experimental protocols were approved by the Tokyo University of Science Animal Care and Use Committee (Permit Number: N12018).

### Luciferase reporter assay

Luciferase reporter gene plasmids driven by the rat BCAM promoter were constructed as follows. Rat BCAM promoters were amplified by PCR using K2 cell genomic DNA and introduced into pGL4.10 vector (Promega) and designated as -1942Luc. The plasmids -1281Luc, -705Luc, -208Luc, -61Luc, and -4Luc were generated by cloning the region of -1942Luc extending from −1281, −705, −208, −61, or −4 to +10. The primer sequences used were as follows: 5′-primer for -1942Luc, 5′-CGT CCT AAA ACT CAA CAA TAG CCA AAG-3′, and 3′-primer for -1942Luc, 5′-ATT CCC TGC AGT GGC GGC AG-3′. For each transfection, 1×10^4^ cells/96-well plate were transfected with 30 ng of each pGL4.10 luciferase reporter vector, 3 ng of Renilla luciferase expression vector pGL4.74, and a total of 10 ng of pcDNA3 empty vector and pcDNA3-FBI1 in various combinations. Twenty-four hours after transfections, cells were lysed with the lysis buffer of the Dual-Luciferase Reporter Assay System (Promega), and luciferase activities were determined according to the manufacturer's instructions using an ARVO Light (Perkin Elmer). Reporter gene activities were normalized using Renilla luciferase activity as an internal control.

### ChIP analysis

Cells were cross-linked with 1% formaldehyde for 10 min. The nuclear fraction was isolated and sonicated to shear genomic chromatin. Chromatin extracts were precleaned with protein G-Sepharose/salmon sperm DNA beads at 4°C for 3 h and then incubated with anti-FBI1 and anti-14-3-3β antibodies at 4°C overnight. Protein G-Sepharose/salmon sperm DNA beads were then added to the mixture for 3 h, and immunoprecipitated DNA-protein complexes were isolated from beads after several washing steps. Reversal of the cross-linking of chromatin was performed at 65°C for 6 h, and proteinase K digestion was allowed to proceed for 1 h at 55°C. DNA was extracted by the phenol/chloroform method. PCR was carried out on purified DNA using primers corresponding to -309 to +49 of the BCAM promoter region (5′-primer, 5′- CGA TTG CCT GGT GAG ATC CAA GCT CG-3′; 3′-primer, 5′- GCC AGC AGG ACT GCG AGC AAC AG -3′).

### Statistical analysis

All data were expressed as mean ± SE of the indicated number of experiments. The statistical significance of differences between mean values was determined by Student's t test. A value of p<0.05 was considered statistically significant.

## Results

### BCAM selectively inhibits anchorage-independent growth

**Figure 1:**
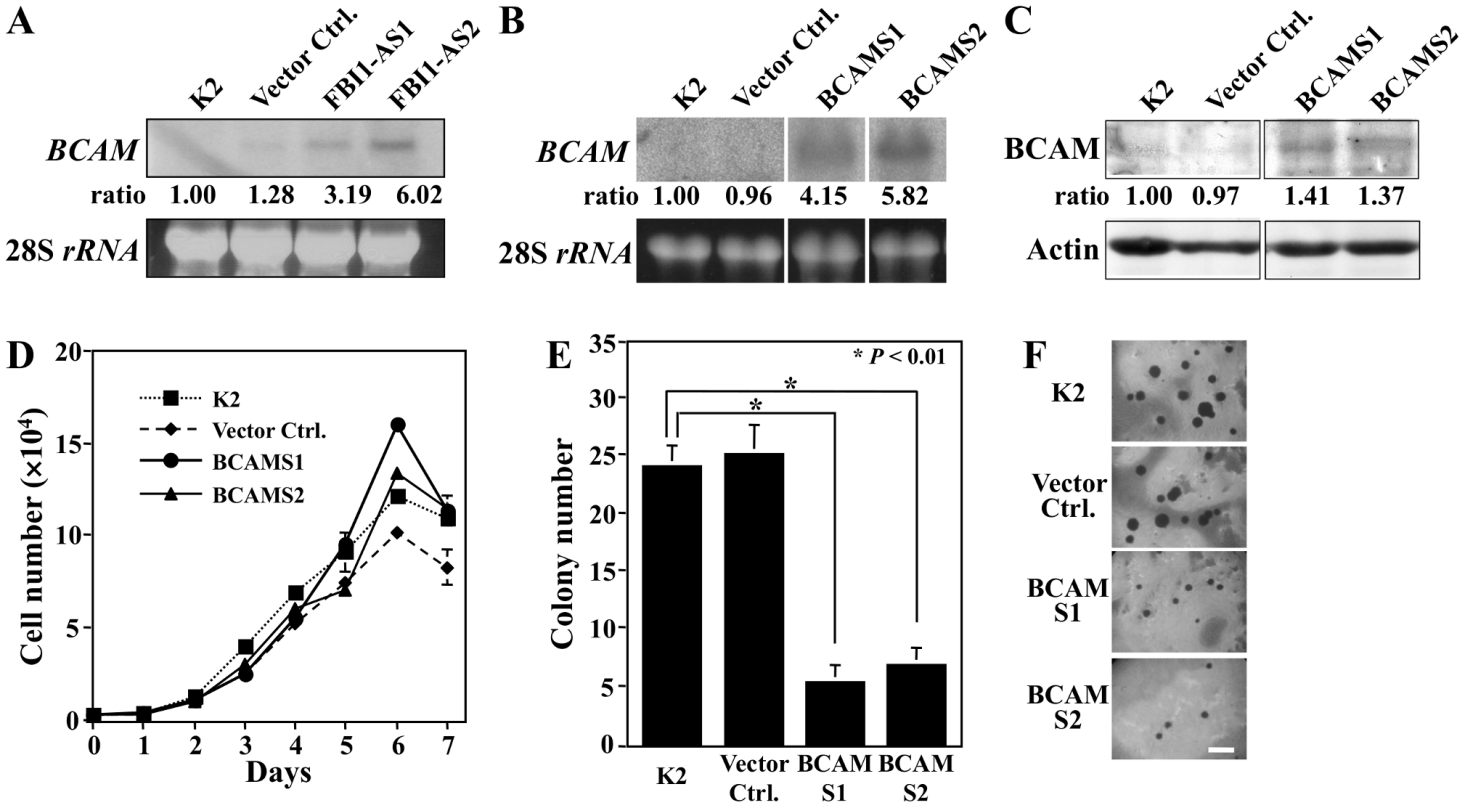
Ectopic expression of BCAM suppresses malignant conversion. (A) BCAM expression is up-regulated in FBI1 knockdown cell lines. Total RNAs were extracted from K2, vector control, and FBI1-AS1/AS1 cells and were analyzed by northern blotting using ^32^P-labeled *BCAM* cDNA as a probe. Ethidium bromide-stained 28S *rRNA* was included for comparison of amount of RNA employed. (B) Characterization of *BCAM* expression plasmid-introduced K2 cells. Total RNAs from K2, vector-introduced control, and *BCAM* expression vector-introduced BCAMS1/S2 cells were analyzed by northern blotting using ^32^P-labeled *BCAM* cDNA as a probe. (C) Expression of BCAM proteins was analyzed by western blotting with anti-BCAM antibody. (D) BCAM does not inhibit substrate-dependent growth. K2, vector control, and BCAM-S1/S2 cells were cultivated for various times and cell numbers were counted. (E, F) BCAM inhibits colony formation of K2 cells in soft-agar medium. K2, vector control, and BCAM-S1/S2 cells were cultivated for 2 weeks in soft-agar medium and photographed after staining with 1-*p*-iodophenyl-*p*-nitrophenyl-5-phenyltetrazolium chloride (INT), and the number of colonies (>0.2 mm in diameter) was counted. *Significant difference from K2 cells (P<0.01), n = 3. Scale bar represents 500 µm.

A cDNA microarray analysis consisting of 23,000 mouse genes revealed that 26 gene expression levels were altered by over two-fold between FBI1-downregulated FBI1-AS1 and parental K2 cells [8]. Among those genes, we chose *BCAM* as one that is possibly suppressed by the 14-3-3β-FBI1/Akirin2 complex for further functional analysis in malignant progression of K2 cells. In order to confirm the microarray data, the expression levels of *BCAM* transcripts in K2 cells, and in vector control and antisense FBI1-introduced FBI1-AS1/AS2 cells [7], were analyzed by northern blotting. Expression levels of *BCAM* mRNA in FBI1-AS1 and FBI1-AS2 cells were 3.2 and 6.0-fold higher than those in the parental K2 cells, respectively (Figure 1A). Thus, this result confirmed the microarray data and raised the possibility that the 14-3-3β-FBI1/Akirin2 complex negatively regulates *BCAM* gene expression.

In order to analyze the function of BCAM protein in tumor cell growth, we introduced a BCAM cDNA expression vector into K2 cells. To confirm the expression levels of BCAM mRNA and protein in transfectants, they were analyzed by northern blotting and western blotting, respectively. *BCAM* mRNA expression levels in BCAMS1 and S2 cells were 4.15 and 5.82-fold higher than that in the parental K2 cells (Figure 1B). The expression levels of BCAM protein in both BCAMS1 and S2 cells were increased to 141 and 137% compared with that of the parental K2 cells, respectively (Figure 1C). Among these cells, differences in the growth rates in monolayer culture were not observed (Figure 1D). Next, these transfectants were cultured in soft agar medium containing 5% FCS for 2 weeks, and the number of colonies (>0.2 mm in diameter) was counted. In contrast, the colony-forming abilities of BCAMS1 and BCAMS2 cells were greatly reduced to 21.9% and 28.3% compared with that of the parental K2 cells, respectively (Figure 1E and F). These results imply that in K2 cells, BCAM selectively inhibits anchorage-independent growth as a hallmark of tumors *in vitro*.

### Suppression of tumorigenicity by forced expression of BCAM

**Figure 2:**
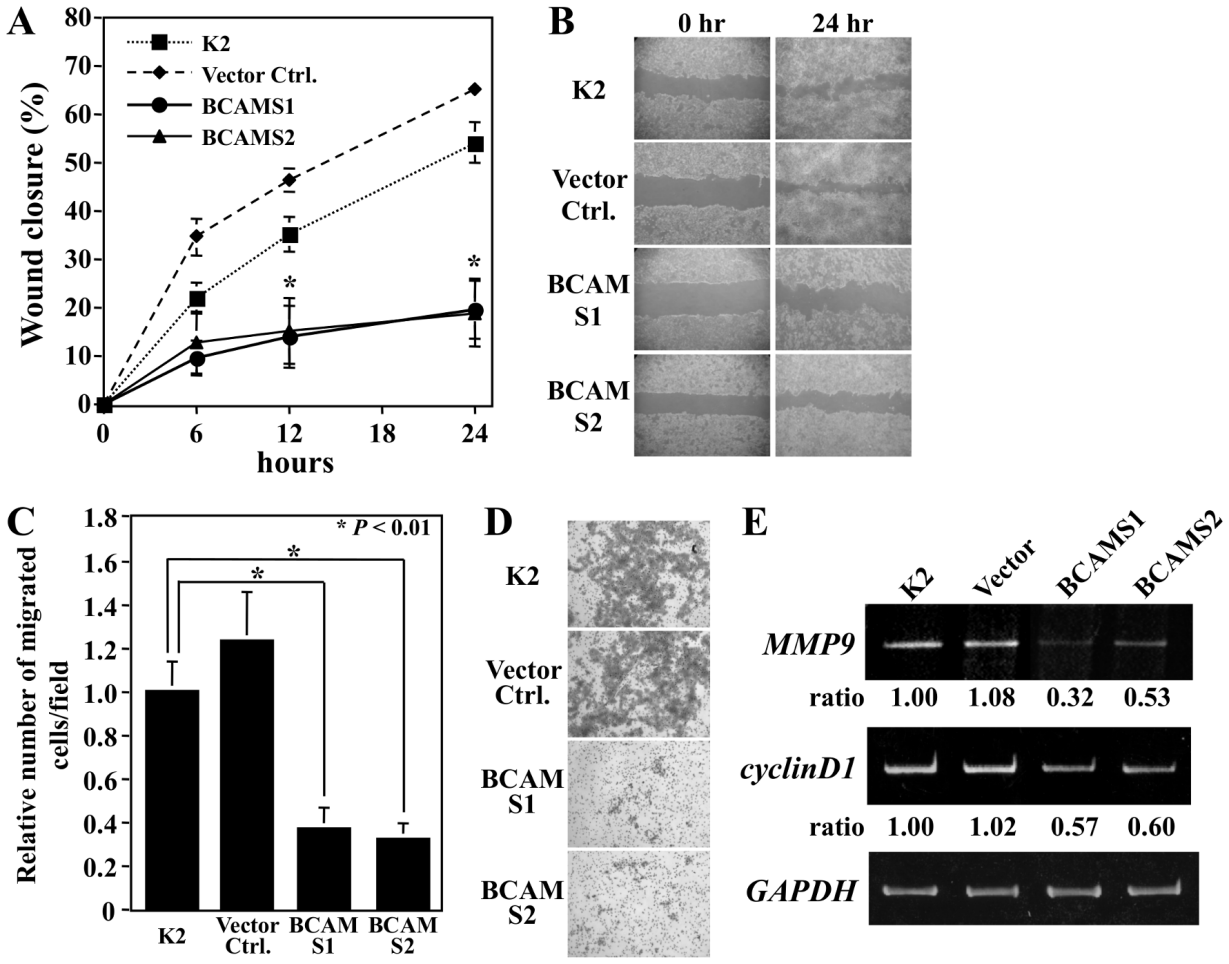
BCAM inhibits cell motility and invasion. (A, B) Wound healing assay for the migration of K2, vector control, and BCAM-S1/S2 cells. The percentage of wound healing was calculated as (width of wound at 0 h - width of wound at 6, 12, 18, or 24 h)/ width of wound at 0 h. *Significant difference from K2 cells (P<0.01), n = 3. (C and D) Invasion potencies of K2, vector control, and BCAM-S1/S2 cells were assayed by a Matrigel-coated chamber plate. Cells (6×10^4^ cells) were seeded and incubated for 48 h. Invading cells on the lower surface of the membrane were photographed (D), and the cell number was counted (C) after staining with crystal violet. *Significant difference from K2 cells (P<0.01), n = 3. (E) BCAM inhibits expression of matrix metalloprotease 9 and cyclin D1. The expression level of MMP-9 and cyclin D1 mRNA was analyzed by RT-PCR. Cyclin D1 mRNA was prepared after 60 min of serum stimulation. Glyceraldehyde 3-phosphate dehydrogenase (GAPDH) mRNA expression was also analyzed as an internal control.

Cancer cell migration and invasion are directly related to metastasis. To examine the effect of BCAM on cell motility and invasion, an *in vitro* wound-healing assay and a cell invasion assay were performed. BCAM transfectants showed 19% and 19.8% wound closure at 24 h, while parental cells and vector control cells showed 54.1% and 65.1%, respectively, suggesting that overexpression of BCAM decreased the migration of K2 cells (Figure 2A and B). Moreover, Matrigel invasion assays were also performed. The numbers of cells that passed through the filter among BCAMS1 cells (22.7±5.9) and BCAMS2 cells (19.8±4.2) were remarkably less than that in the parental cells (61.6±8.6) or vector control cells (76.2±13.8), which shows that upregulation of BCAM expression suppressed K2 cell invasion *in vitro* (Figure 2C and D).

Matrix metalloproteinases (MMPs) degrade extracellular matrix, and the expression level of MMPs is correlated with the metastatic ability of cancer cells [17]–[20]. In particular, the activities of MMP-2 and MMP-9 are often found to be elevated in tumor tissues and malignant cancer cells. Therefore, we analyzed the expression levels of *MMP-2* and *MMP-9* by RT-PCR. The expression levels of *MMP-9* mRNA in BCAMS1 and BCAMS2 cells were strongly diminished compared with levels in K2 and vector control cells, whereas altered expression of *MMP-2* was not observed (Figure 2E). Also, cyclin D1 is overexpressed in many cancers, and sustained ERK activation leads to the induction of cyclin D1. In addition, FBI1/Akirin2 can promote cell growth by extending the duration of ERK1/2 activation [7]. The expression level of cyclin D1 was analyzed by RT-PCR. In BCAM transfectants, the expression of cyclin D1 was significantly reduced compared with that of K2 and vector control cells (Figure 2E).

**Figure 3:**
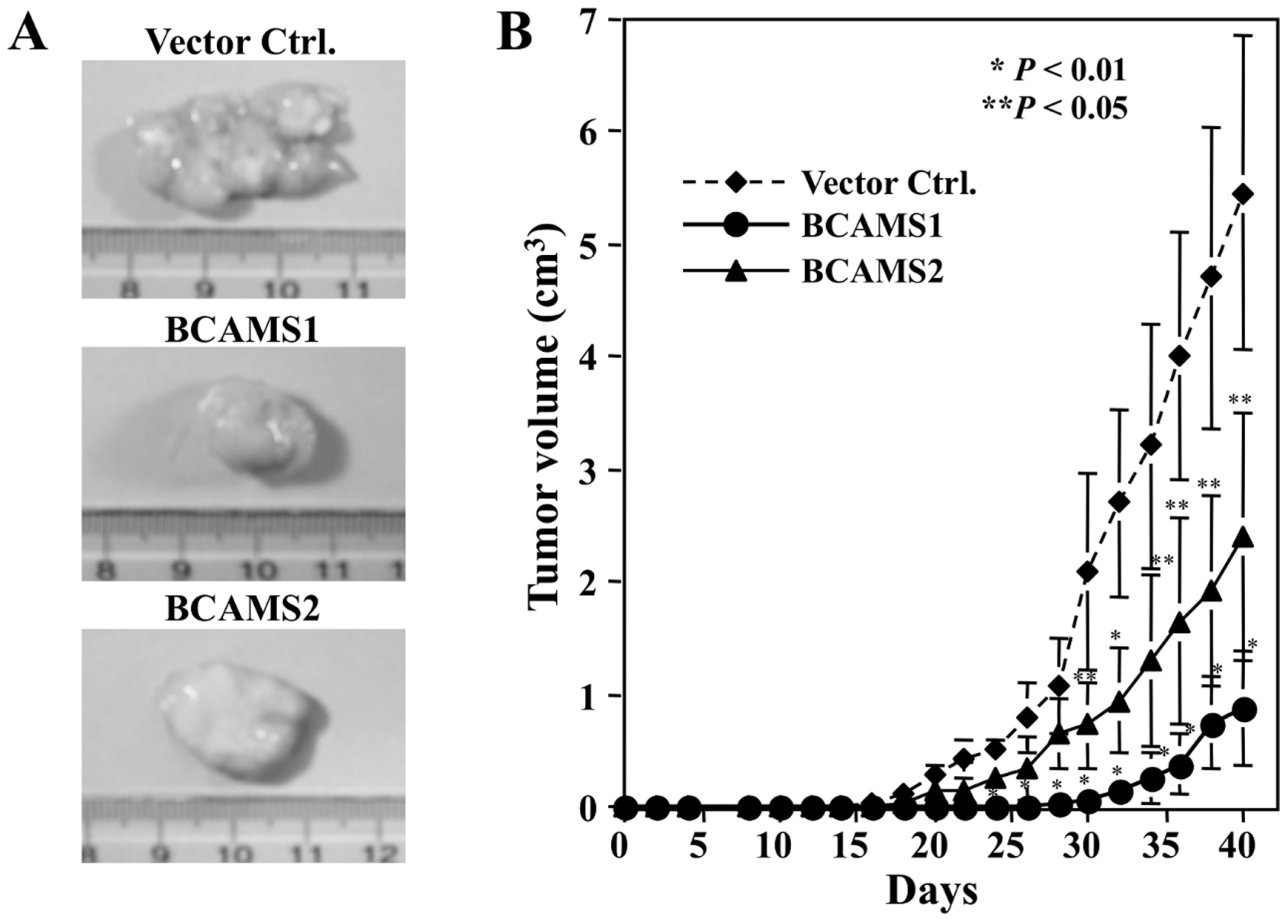
Ectopic expression of BCAM suppresses tumorigenicity of K2 cells. Vector control and BCAMS1/2 K2 transfectans were subcutaneously injected into the flanks of SCID mice (5×10^4^ cells/animal). After 40 days, the tumors formed were photographed (A) and tumor volumes were measured every other day (B). Significant differences from vector control cells were *P<0.01 and ** P<0.05.

Moreover, to investigate effects of overexpression of BCAM on the tumorigenicity of K2 cells, the transfectants were subcutaneously inoculated into the flank of NOD/SCID mice, and the sizes of the tumors were measured every 2 days. Forty days after subcutaneous injection into NOD/SCID mice, vector cells formed tumors with a mean volume of 5.46 cm^3^, whereas BCAMS1 and BCAMS2 cells formed smaller tumors with mean volumes of 2.41 and 0.87 cm^3^, respectively (Figure 3A and B). These results show that forced expression of BCAM inhibits the tumorigenicity and metastasis of K2 cells.

### The BCAM cytoplasmic domain regulates tumor suppression

**Figure 4:**
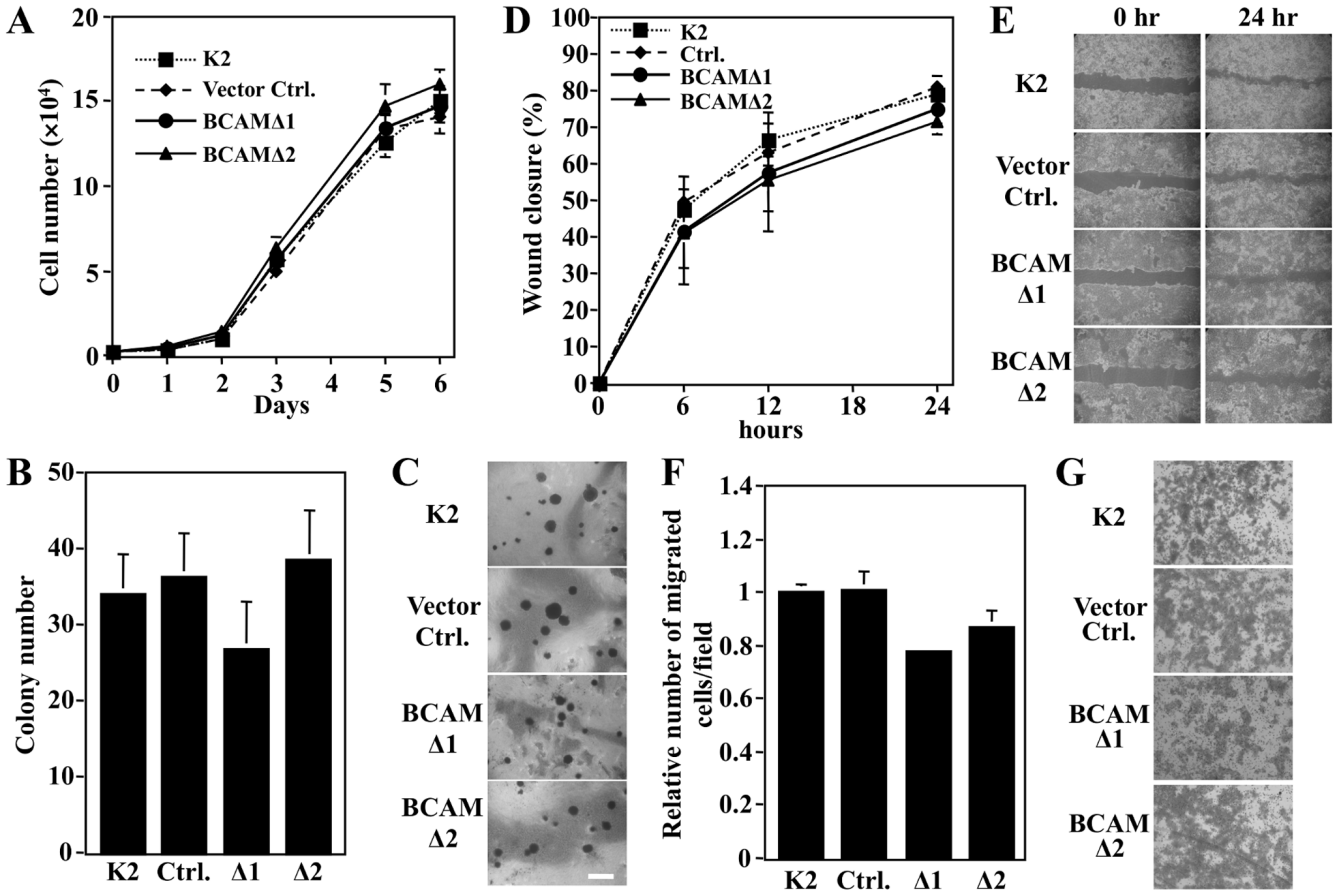
BCAM cytoplasmic domain deletion mutant does not influence tumor suppression. A deletion mutant expression vector of the cytoplasmic domain of BCAM was introduced into K2 cells. (A) BCAM does not inhibit substrate-dependent growth. K2, vector control, and BCAM-Δ1/Δ2 cells were cultivated for various times and the cell number was counted. (B and C) BCAM does not inhibit colony formation of K2 cells in soft-agar medium. K2, vector control, and BCAM-Δ1/Δ2 cells were cultivated for 2 weeks in soft-agar medium and photographed after staining with INT, and the number of colonies (>0.2 mm in diameter) was counted. Scale bar represents 500 µm. (D and E) Wound healing assay for the migration of K2, vector control, and BCAMΔ1/Δ2 cells. The percentage of wound healing was calculated described in the legend of Fig. 2. (F and G) Invasion potencies of K2, vector control, and BCAM-Δ1/Δ2 cells were assayed with Matrigel-coated chamber plates. Cells (6×10^4^ cells) were seeded and incubated for 48 h. Invading cells on the lower surface of the membrane were photographed (G) and the cell number was counted (F) after staining with crystal violet.

The cytoplasmic domain of BCAM possesses sites for phosphorylation by glycogen synthase kinase 3β (GSK-3β), casein kinase II (CKII), and PKA [21], suggesting possible signal-transduction functions. Therefore, to investigate whether the cytoplasmic domain of BCAM is essential for tumor suppression, we established a BCAM cytoplasmic domain deletion mutant (Δ571– 624) expression vector and introduced it into K2 cells. In monolayer culture, there were no differences in growth rates among these cells as with BCAM transfectants (Figure 4A). However, for the colony forming ability of BCAMΔ1 and BCAMΔ2 transfectants in soft agar, there was no significant difference observed (Figure 4B and C). In addition, BCAMΔ1 and BCAMΔ2 transfectants did not influence either migration (Figure 4D and E) or invasion (Figure 4F and G). These results imply that the BCAM cytoplasmic domain plays a pivotal role in the inhibition of malignant progression of K2 cells.

### The 14-3-3β-FBI1/Akirin2 complex suppresses BCAM promoter activity

**Figure 5:**
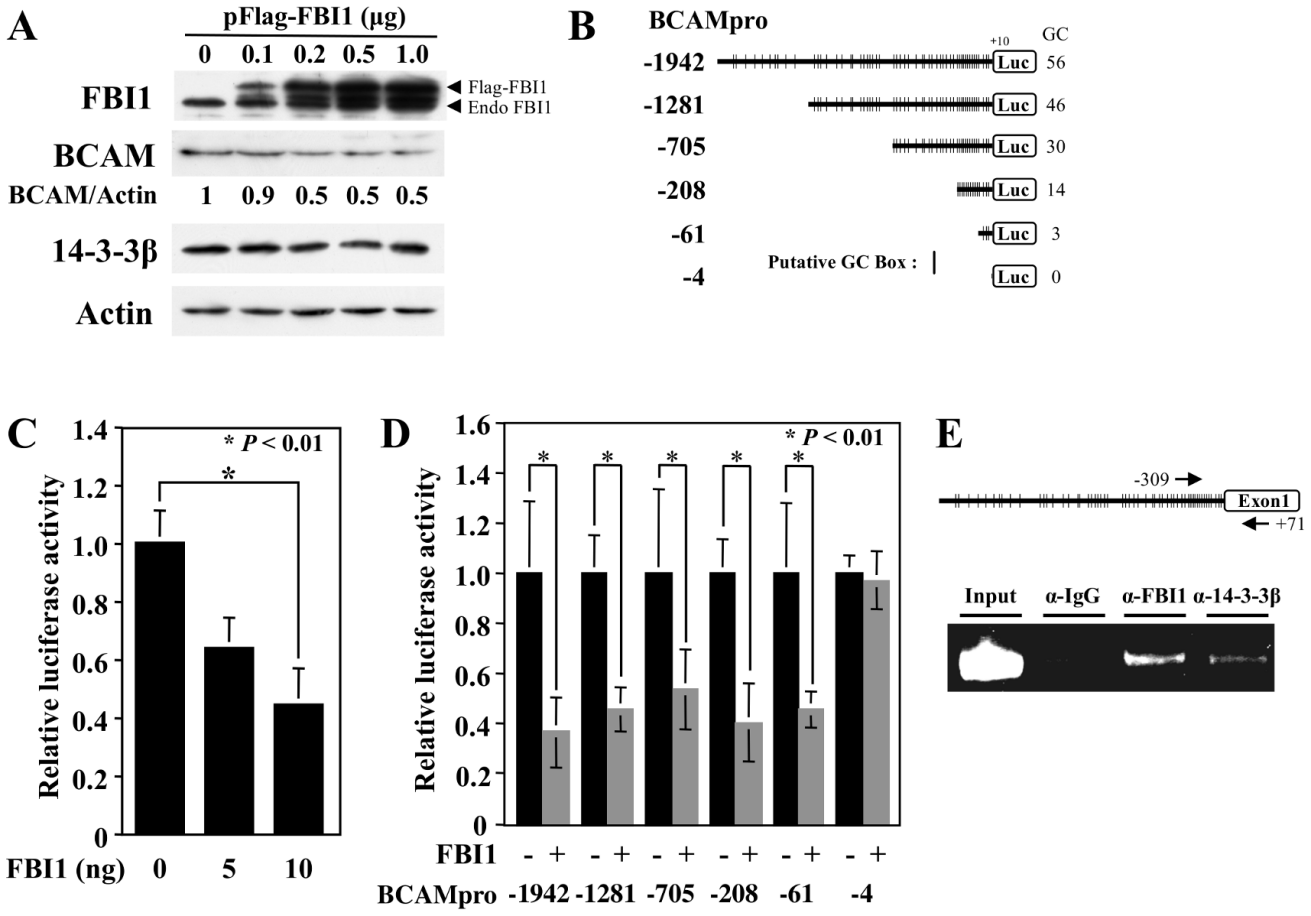
The 14-3-3β-FBI1/Akirin2 complex suppresses BCAM promoter activity. (A) Stimulation of BCAM protein expression by ectopic expression of FBI1/Akirin2. Cultured K2 cells were transfected with various amounts of pFlag-FBI1, and after 24 h, BCAM protein was analyzed by WB. (B) Schematic illustration of rat *BCAM* promoter-linked luciferase reporter plasmids. (C) FBI1/Akirin2 suppresses the promoter activity of pBCAMpro-1942 reporter plasmid. K2 cells were co-transfected with 30 ng pBCAMpro-1942 and a total of 10 ng pcDNA3-FBI1 and pcDNA3 in the indicated ratio together with 3 ng Renilla luciferase vector pGL4.75 as an internal control by lipofection. 24 h after transfection, luciferase activities were measured using a Dual Luciferase Assay Kit. Each value indicates the mean ± S.E. Comparisons of data were performed by Student's *t*-test. *P<0.01, significantly different from vector-introduced control cells, *n* = 3. (D) The proximal GC box is required for downregulation of *BCAM* expression. Different promoter constructs were introduced into K2 cells under the presence or absence of 10 ng pcDNA3-FBI1 expression vector. 24 h after transfection, luciferase activities were measured as described above. (E) FBI1/Akirin2 suppresses BCAM expression via the transcriptional suppressor complex. Soluble chromatins were immunoprecipitaed with anti-FBI1 or anti-14-3-3β and analyzed by PCR using a primer set covering the -309 to +71 promoter region as indicated by arrows.

Previously, we reported that the 14-3-3β-FBI1/Akirin2 complex represses *MKP-1* transcription via binding with the GC box in the promoter region [7]. Therefore, to examine whether the 14-3-3β-FBI1/Akirin2 complex regulates *BCAM* gene expression, K2 cells were transfected with various amounts of Flag-FBI1 expression vector, and after 24 h expression levels of BCAM protein were analyzed. BCAM protein expression was dose-dependently downregulated by the ectopic expression of FBI1, up to a maximum of 0.2 µg of FBI1, at which point an approximately 50% reduction was observed (Figure 5A). To further investigate the suppressive effect of BCAM on FBI1/Akirin2 expression, we constructed the luciferase reporter gene plasmid pBCAMpro-1942, which is driven by the putative 56 GC boxes-existing region of the *BCAM* promoter from -1942 to +10 (Figure 5B). To analyze the effect of FBI1/Akirin2 on *BCAM* promoter activity, pBCAMpro-1942 and various amounts of FBI1/Akirin2 expression vector pcDNA3-FBI1 were co-transfected with K2 cells, and after 24 h luciferase activities were determined. FBI1/Akirin2 suppressed the *BCAM* promoter activity in a dose-dependent manner, which was reduced to 48% of the control when co-transfected with 10 ng of FBI1 expression plasmid (Figure 5C), indicating that FBI1/Akirin2 is a rate-limiting factor for the formation of the repressive transcription complex. To identify which GC box is required for the repression, different *BCAM* promoter constructs were introduced into K2 cells together with 10 ng of pcDNA3-FBI1, and were assayed for luciferase activities (Figure 5B and D). pBCAMpro-61, which contains the proximal three GC boxes, showed repressed luciferase activity similar to levels observed in pBCAMpro-1942/-1281/-705/-208, while pBCAMpro-4, which possesses no GC box, was unaffected (Figure 5D), indicating that FBI1/Akirin2 suppressed BCAM promoter activity via the proximal three GC boxes. Finally, to confirm whether the 14-3-3β-FBI1/Akirin2 complex binds to the BCAM promoter region *in vivo*, we carried out ChIP analysis using anti-14-3-3β and anti-FBI1 antibodies. These factors were detected in the -309 to +71 *BCAM* promoter region (Figure 5E), showing that the 14-3-3β-FBI1/Akirin2 complex suppresses *BCAM* gene expression via the proximal three GC boxes, at least in part. This is the first demonstration of transcriptional suppression of the *BCAM* gene by the 14-3-3β-FBI1/Akirin2 complex.

## Discussion

In this study, we showed that downregulation of BCAM by the 14-3-3β-FBI1/Akirin2 complex is also implicated in malignant conversion of K2 cells. AFB_1_ causes genetic alterations leading to deregulated expression of genes such as *14-3-3*β and *FBI1/Akirin2* during hepatocarcinogenesis. Over-expressed 14-3-3βand FBI1/Akirin2 form the 14-3-3β-FBI1/Akirin2 transcriptional repressor complex that restrains the expression of the suppressive oncogene *BCAM* via association with the GC box in the promoter region. Downregulation of BCAM confers potent anchorage-independent growth, cell migration, invasion, and tumorigenicity.

Basement membrane laminin plays an active role in regulating the migration, proliferation, and progression of malignant tumors through its interaction with specific cell surface receptors [9]. BCAM deficient cells do not bind to laminin, whereas overexpression of BCAM increases binding to laminin [22]. Therefore, it is thought that BCAM plays a role in transmitting the signal from the extracellular matrix into the cell. In addition, the cytoplasmic domain of BCAM is phosphorylated by GSK-3β, CKII, and PKA at serines 596, 598, and 621, respectively [21]. Mutation of serine 621 prevents phosphorylation by PKA and dramatically decreases cell adhesion to laminin α5 [21]. In this way, the phosphorylation of the BCAM cytoplasmic domain by PKA plays an important role in cell attachment. Our results revealed that the deletion of the BCAM cytoplasmic domain, which contains the phosphorylation sites, resulted in lost inhibition of malignant conversion. Though more study is needed, our results indicate phosphorylation of the BCAM cytoplasmic domain plays an important role in tumor suppression.

Luciferase reporter and ChIP analyses revealed that the 14-3-3β-FBI1/Akirin2 complex can act as a transcriptional suppressor of *BCAM.* We previously reported that the 14-3-3β/FBI1/Sp3/HDAC1 complex promotes sustained ERK1/2 activation through repression of *MKP-1* transcription, leading to promotion of tumorigenicity and metastasis of K2 cells [7]. Several reports have demonstrated that Akirin proteins regulate transcription. Akirin2 is required for NF-κB-dependent gene expression induced by the Toll-like receptor, tumor necrosis factor, and interleukin-1β signaling [23]. For regulation of NF-κB by Akirin2, it is necessary to form a complex involving such factors as DNA-binding proteins, transcriptional activators or repressors, or chromatin-remodeling proteins, because Akirins do not bind to DNA or NF-κB by themselves [23]–[25]. Therefore, Akirin2 may regulate the expression of various genes by differences in its binding partners.

BCAM antigen was first identified by monoclonal antibodies raised against human tumor cells [26], and BCAM has an amino acid sequence with structural homology to MUC18, a human metastatic melanoma cell surface antigen [27]. In addition, BCAM overexpression has previously been described in epithelial skin tumors [9], [10], [28], ovarian cancer [29], pancreatic cancer [30], and hepatocellular carcinoma [11]. These above-mentioned results contradict our results. However, these reports showed only the expression level of BCAM in tumors, whereas in the present report, we demonstrated for the first time a function of BCAM in which it acts as a suppressive oncogenic protein. Furthermore, other reports have shown a significant reduction on BCAM expression in metastatic colon cancer cell lines [31] and malignant thyroid cancer [32]. The BCAM of the laminin receptor is presumed to play a potential role in malignant tumors.

The 14-3-3 family proteins are ubiquitously expressed and are phosphoserine/threonine specific binding proteins, and interact with many proto-oncogene and oncogene products [1]. Furthermore, the FBI1/Akirin2 gene was robustly expressed in rat HCCs, glioblastoma cells, pheochromocytoma cells, and embryonic carcinoma cells, compared with the respective normal tissues [7]. On the other hand, the expression level of FBI1/Akirin2 in non-tumor cell lines, such as COS-7, Balb3T3, and REF52 cells, was very low [7]. Therefore, it is thought that overexpression of FBI1/Akirin2 is implicated in malignant transformation via suppression of such as BCAM and/or MKP-1. In addition, FBI1/Akirin2 suppressed the *BCAM* promoter activity in a variety of cell lines such as rat glioma C6, Huh7, HeLa and COS-7, by luciferase reporter assay (Figure S1). Consequently, it is sure that FBI1/Akirin2 regulates expression of BCAM in various cell lines. Nonetheless, the expression level of BCAM varies among the cancer cell lines [12], [27]. The BCAM expression in human HCC was found in HepG2 and Huh7, but not in HLE and HLF [12]. In our results, the BCAM expression was not found in rat HCCs, such as K2, AH-60, AH-70 and dRLa-84, except for dRLa-74 by northern blotting (Figure S2). Thus, it is not revealed whether this pathway is common because BCAM expression levels are different among HCC cell lines. In conclusion, our results suggest that BCAM contributes to the suppression of malignant progression in K2 cells.

## Supporting Information

Figure S1 **FBI1/Akirin2 suppresses BCAM promoter activity in various cell lines.** K2, C6, Huh7, HeLa and COS-7 cells were co-transfected with 30 ng pBCAMpro-1942 and presence or absence of 10 ng pcDNA3-FBI1 expression vector in the indicated ratio together with 3 ng Renilla luciferase vector pGL4.75 as a internal control by lipofection. 24 h after transfection, luciferase activities were measured using a Dual Luciferase Assay Kit. Each value indicates the mean ± S.E. Comparisons of data were performed by Student's *t*-test. *P<0.01, significantly different from vector-introduced control cells, *n* = 8.
(TIF)

Figure S2 **Expression levels of** ***FBI1/Akirin2*** **mRNA in various cell lines.** Total RNAs were extracted from various cell lines, including rat hepatocarcinoma cells (K2, AH60, AH70, dRLa-74, dRLa-84 and K1), rat glioblastoma cells (C6), embryonic carcinoma cells (P19), human hepatocarcinoma cells (HepG2), cervical carcinoma cells (HeLa), and normal prostate tissue analyzed by northern blotting using ^32^P-labeled *BCAM* cDNA as a probe.
(TIF)
